# Beyond Simple Ratios: Can the Preoperative HALP Score Provide a More Effective Inflammatory Biomarker for Colon Cancer Survival?

**DOI:** 10.3390/medicina62091627

**Published:** 2026-08-25

**Authors:** Abdullah Durhan, Yusuf Murat Bağ, Mehmet Ali Çaparlar, İbrahim Kurtoğlu, Yasin Uçar, Koray Koşmaz, Abdullah Bulgurcu, Marlen Süleyman

**Affiliations:** 1 Department of Surgical Oncology, Ankara Training and Research Hospital, 06030 Ankara, Türkiye; 2 Department of General Surgery, Polatlı Duatepe State Hospital, Ministry of Health, 06430 Ankara, Türkiye

**Keywords:** colon cancer, HALP score, inflammatory biomarkers, nutritional status, prognosis, overall survival

## Abstract

*Background and Objectives*: Previous findings have suggested that inflammatory and nutritional biomarkers may help predict the prognosis in colorectal cancer patients; however, their predictive value and optimal use remain uncertain. Therefore, this study aimed to investigate the effects of several preoperatively measured inflammatory and nutritional biomarkers on the overall survival in patients undergoing surgery for colon cancer and to compare their predictive ability between themselves and with other clinical parameters. *Materials and Methods*: This retrospective cohort study included 411 patients who underwent surgery for colon cancer between May 2014 and May 2024. Demographic, clinical, pathological, and laboratory data were analyzed. The neutrophil-to-lymphocyte ratio (NLR), platelet-to-lymphocyte ratio (PLR), De Ritis ratio, Gustave Roussy Immune (GRIM) score, and hemoglobin, albumin, lymphocyte, and platelet (HALP) score were calculated from preoperative laboratory values. Cox regression, receiver operating characteristic (ROC), and Kaplan–Meier survival analyses were performed. *Results*: During follow-up, 270 patients (65.7%) survived, and 141 (34.3%) died. The survival group was significantly younger than the mortality group (*p* < 0.001). The hemoglobin level and HALP score were significantly higher in the survival group, whereas the PLR was significantly higher in the mortality group. In the multivariate analysis, adjusted for baseline clinical and pathological factors, the age (HR: 1.046, 95% CI: 1.030–1.062, *p* < 0.001) and the HALP score (HR: 0.984, 95% CI: 0.971–0.998, *p* = 0.025) were identified as independent predictors of mortality. A HALP score cut-off value of 38.8 predicted mortality with 85.1% sensitivity and 31.9% specificity (AUC: 0.597, *p* = 0.001). Patients with HALP scores > 38.8 had significantly longer overall survival (*p* < 0.001). *Conclusions*: The preoperative HALP score is an independent predictor of overall survival in patients with colon cancer. As a simple and readily available immune–nutritional biomarker, the HALP score may contribute to preoperative risk stratification and prognostic assessment in routine clinical practice, serving as a supplementary tool alongside traditional staging.

## 1. Introduction

Among gastrointestinal malignancies, colon cancer ranks among the most frequent and remains a major contributor to cancer-related mortality worldwide, accounting for more than 1.9 million new diagnoses and close to 930,000 deaths each year [1]. Its incidence continues to climb in Western nations and in rapidly developing economies, a trend linked to shifting dietary habits, population aging, and increasingly sedentary lifestyles over the last decade [2]. Although screening methods, diagnostic techniques, and treatment strategies have improved considerably, outcomes for locally advanced or metastatic tumors remain unsatisfactory [3], reflecting the combined influence of tumor stage, histopathological characteristics, and molecular subtype [4]. Notably, survival can vary substantially even among patients who share an identical pathological stage, underscoring the need for biological markers that extend beyond conventional anatomical staging [5]. Accumulating evidence further indicates that both nutritional status and systemic inflammatory activity meaningfully influence these outcomes [6].

The immune system normally exerts an antitumorigenic effect; however, once inflammation becomes protumorigenic, it can promote malignancy by suppressing antitumor immune responses and remodeling the tumor microenvironment toward tumor-favorable signaling [7]. Consequently, how inflammation shapes cancer development, progression, and treatment response has drawn considerable research interest [7]. At the same time, malnutrition is known to worsen clinical outcomes and reduce survival among cancer patients [8]. For this reason, a range of inflammatory and nutritional biomarkers has been proposed as potential prognostic indicators in colorectal cancer [9]. Integrating multiple hematological and biochemical parameters, rather than relying on a single measure such as the white blood cell count alone, may offer a more complete picture of the host-tumor interaction [10].

Previous findings have underlined that taking into account inflammatory and nutritional scores and biomarkers may help predict the prognosis and improve the survival rate in patients with colorectal cancer, but this needs confirmation and standard cut-off values [9,11]. Among these composite indices, the Hemoglobin, Albumin, Lymphocyte, and Platelet (HALP) score has gained particular attention [12]. The HALP score combines four parameters that each reflect a different aspect of host status: hemoglobin (tissue oxygenation), albumin (nutritional and acute-phase status), lymphocytes (cell-mediated immunity), and platelets (coagulation and tumor-promoting inflammation). This combination may give a more complete picture of the patient’s overall immune and physiological competence compared to simpler two-cell ratios like the neutrophil-to-lymphocyte ratio (NLR) or platelet-to-lymphocyte ratio (PLR) [13].

A major obstacle to using the HALP score in routine practice is the lack of a standardized measurement unit, which produces widely different cut-off values across studies [13]. Therefore, the present study was designed to investigate the effects of several preoperatively measured inflammatory and nutritional biomarkers on the 10-year overall survival in patients undergoing elective surgery for colon cancer, and to compare their predictive ability between themselves and with other clinical parameters.

## 2. Materials and Methods

This was a retrospective cohort study. Ethical approval was obtained from the local ethics committee (approval number: 24:159, dated 6 June 2024). The data of patients with colon cancer who were operated on in the general surgery and surgical oncology department of the tertiary hospital between May 2014 and May 2024 were retrieved from the electronic database. The inclusion criteria were patients aged between 18 and 100 years, having undergone surgery with a colon cancer diagnosis (open and minimally invasive surgery), and who had data availability.

**Figure 1:**
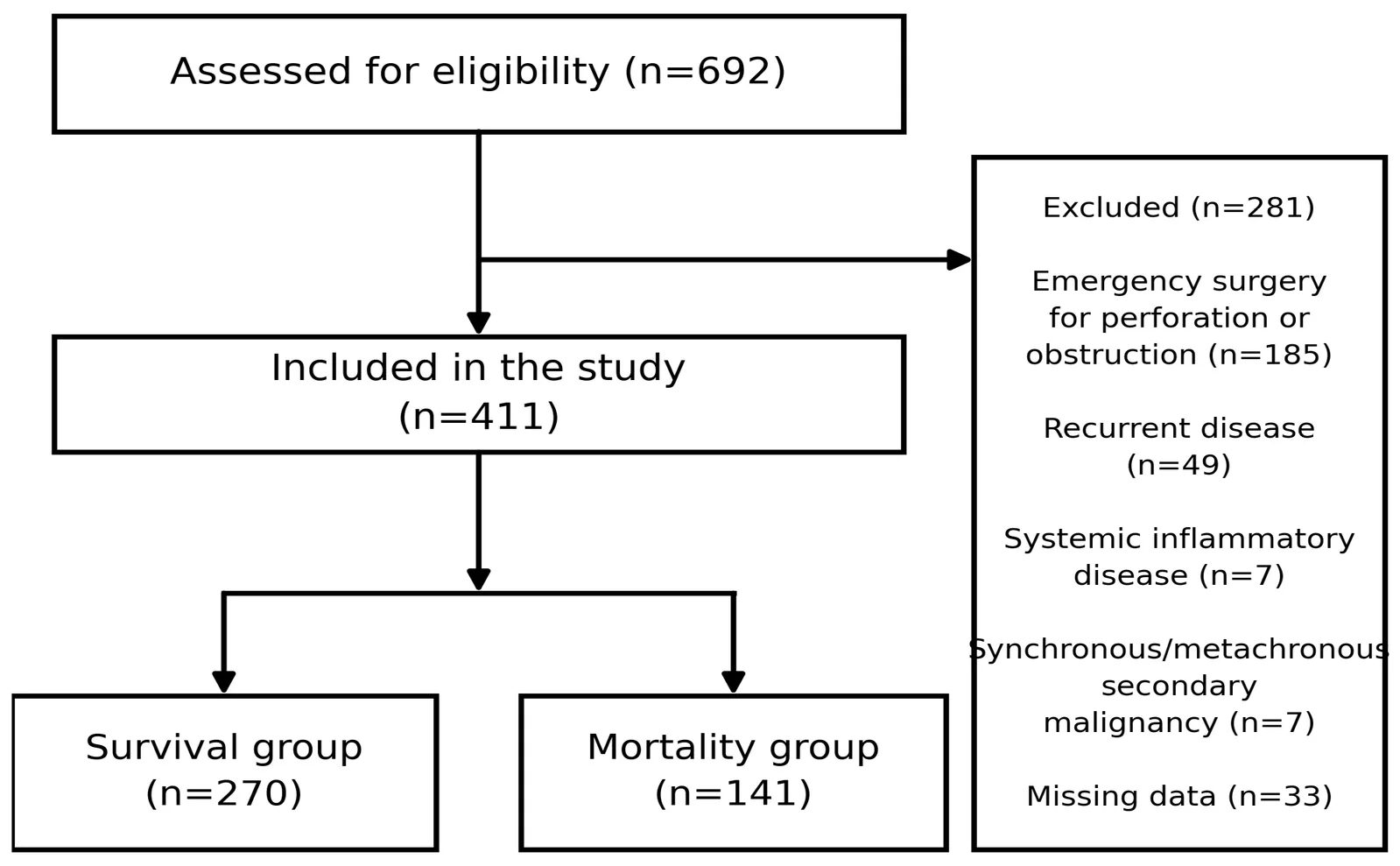
CONSORT-style flow diagram illustrating patient screening and selection. Of 692 patients assessed for eligibility, 281 were excluded (emergency surgery for perforation or obstruction, *n* = 185; recurrent disease, *n* = 49; systemic inflammatory disease, *n* = 7; synchronous or metachronous secondary malignancy, *n* = 7; missing data, *n* = 33), yielding a final cohort of 411 patients (270 in the survival group and 141 in the mortality group).

Patients who underwent emergency surgery for perforation or obstruction were excluded, since the acute inflammatory response in these settings alters baseline hematological and biochemical markers. Recurrent colon cancers were also excluded, as they differ biologically from primary tumors and often involve prior treatment exposure. Additional exclusion criteria were active systemic inflammatory disease (e.g., rheumatoid arthritis, ankylosing spondylitis), synchronous or metachronous secondary malignancies, and missing data. In total, 411 patients were included in the study. Patient screening and selection are detailed in the CONSORT-style flow diagram (Figure 1).

Demographics (age, gender), laboratory data [indirect bilirubin (mg/dL), direct bilirubin (mg/dL), total bilirubin (mg/dL), lactate dehydrogenase (LDH (U/L)), albumin (g/L), total protein (g/L), alanine aminotransferase (ALT (U/L)), aspartate aminotransferase (AST (U/L)), carcinoembryonic antigen (CEA (ng/mL)), cancer antigen 19-9 (CA 19-9 (U/mL)), neutrophil count (10^9^/L), lymphocyte count (10^9^/L), platelet count (10^9^/L), and hemoglobin (g/L)], and clinical data (tumor location, length of hospital stay, survival status, and survival time) were analyzed.

Several inflammatory and nutritional indices were calculated based on the preoperative laboratory values. The neutrophil-to-lymphocyte ratio (NLR) was calculated as the neutrophil count (10^9^/L)/lymphocyte count (10^9^/L), and the platelet-to-lymphocyte ratio (PLR) was calculated as the platelet count (10^9^/L)/lymphocyte count (10^9^/L). The De Ritis ratio was calculated as the AST level/ALT level [14]. The Gustave Roussy Immune (GRIM) score was calculated using three laboratory parameters: albumin (≥35 g/L = 0 point, <35 g/L = 1 point), LDH (≤250 U/L = 0 point, >250 U/L = 1 point), and NLR (≤6 = 0 point, >6 = 1 point), for a total of 0–3 points [15]. The hemoglobin, albumin, lymphocyte, and platelet (HALP) score was calculated as hemoglobin (g/L) × albumin (g/L) × lymphocyte count (10^9^/L)/platelet count (10^9^/L) [12]. Hemoglobin was expressed in grams per liter (g/L) rather than the more commonly used grams per deciliter (g/dL) throughout the database, to allow direct comparison with international studies. The HALP score was calculated using the following standardized formula:

Hemoglobin (g/L) × Albumin (g/L) × Lymphocyte count (10^9^/L)/Platelet count (10^9^/L).

Pathological data were analyzed based on the American Joint Committee on Cancer (AJCC) TNM Staging Manual, Eighth Edition. The pathological characteristics included the assessment of the primary tumor depth (T stage; Tis, 1, 2, 3, 4), regional lymph node involvement (N stage; 0, 1, 2a, 2b), and the presence of distant metastasis (M stage; 0, 1) [16]. Formalin-fixed, paraffin-embedded tissue blocks and original hematoxylin and eosin-stained slides were retained in our institutional pathology archive to confirm the histological diagnoses.

Preoperatively, patients underwent a colonoscopy, and biopsies were obtained for diagnosis. Multiphasic contrast-enhanced thoracic and abdominopelvic computed tomography was performed for staging. All patients were discussed in a multidisciplinary oncology council, and treatment choices were made by this council. None of the patients underwent neoadjuvant chemotherapy or radiotherapy. Operations were performed by senior surgeons for laparoscopic and open approaches. The operation type depended on the tumor location (for right-sided tumors, right hemicolectomy or extended right hemicolectomy; for left-sided tumors, anterior resection; and for transverse colon tumors, extended right hemicolectomy or left hemicolectomy). All patients were followed up in the intensive care unit for the first day after surgery and were taken to the service when they were stable. Oral intake was given on day 1 with clear liquids and increased gradually if the patient tolerated it. Urinary catheters were removed on postoperative day 1, and abdominal drains were removed when the daily drainage volume fell below 30 mL (<30 mL/24 h). Patients were discharged when they were clinically stable, fully mobilized, had solid oral intake, and no intravenous analgesic was needed. The follow-up was conducted as follows: postoperative first week, first month, and every three months in the first two years. After two years, it was conducted at six-month intervals. At each visit, a clinical history, physical examination, and serum carcinoembryonic antigen (CEA) level were obtained. Surveillance thoracoabdominopelvic computed tomography was performed annually for the first five years, and surveillance colonoscopy was performed at one year after surgery, with the timing of subsequent examinations determined by the findings of that examination.

### Statistical Analysis

IBM SPSS version 25.0 (IBM Corp., Armonk, NY, USA) was used for statistical analyses. The Kolmogorov–Smirnov test was performed for the distribution analysis of continuous variables. As all continuous variables were non-normally distributed, they were expressed as the median (25–75 interquartile range) and were compared using the Mann–Whitney U test. Categorical variables were presented as the frequency (percentage), and the Chi-Square test or Fisher’s Exact test was used for intergroup comparisons. Univariate Cox regression analyses were performed to analyze the independent predictors of overall survival.

The multivariable Cox proportional hazards regression model included age, gender, pathological T stage, pathological N stage, distant metastasis (M stage), and postoperative adjuvant chemotherapy status as forced covariates alongside the inflammatory markers. Pathological parameters were dichotomized to address sub-stage clustering: early T (Tis-T2) versus advanced T (T3–T4), and node-negative (N0) versus node-positive (N1–N2). The proportional hazards assumption was verified using graphical inspection of Log-Minus-Log (LML) survival curves and time-dependent covariate interaction tests (*p* > 0.05). Internal validation was performed using bootstrapping with 1000 resamples to assess model stability. The multivariable results were expressed as the hazard ratio (HR), 95% confidence interval (CI), and *p*-value.

Receiver operating characteristic (ROC) curve analyses were performed for continuous variables that were found to be independent predictors utilizing the Youden Index (Sensitivity + Specificity − 1) to identify optimal mathematical cut-off thresholds. The results were presented as the area under the curve (AUC), 95% CI, *p*-value, cut-off value, sensitivity, and specificity. Kaplan–Meier survival analyses and log-rank tests were performed to examine and compare the overall survival for the variables identified as independent predictors. A two-tailed *p*-value < 0.05 was considered statistically significant.

## 3. Results

The median age of the study group was 65 (56–72) years, and about seven in ten patients were male; the most common tumor location was right-sided across all groups. During the follow-up period, 65.7% (n = 270) of the 411 patients survived and 34.3% (*n* = 141) died. The survival group was statistically significantly younger than the mortality group (63 (53–71) years vs. 69 (62–77) years, *p* < 0.001).

Regarding the laboratory data, preoperative hemoglobin levels, uniformly standardized to grams per liter (g/L), were analyzed alongside the recalculated composite indices. The hemoglobin level was statistically significantly higher in the survival group compared with the mortality group (117 (104–130) g/L vs. 112 (103–123) g/L, *p* = 0.007). In contrast, the PLR was statistically significantly higher in the mortality group (158.73 (129.45–242.52) vs. 156.22 (105.24–222.78), *p* = 0.033). The universally standardized HALP score was found to be statistically significantly higher in the survival group (29.6 (21.3–45.5) vs. 27.0 (17.7–35.0), *p* = 0.001). Finally, the survival time was statistically significantly longer in the survival group as expected (58.08 (39.22–84.31) months vs. 23.23 (9.03–47.89) months, *p* < 0.001). Table 1 shows the demographics, clinical, and laboratory data of the whole study group and the subgroups according to the survival status in patients with colon cancer, additionally including comprehensive cumulative AJCC stage groupings and postoperative adjuvant chemotherapy completion rates.

In the univariate analyses, age (HR: 1.044, 95% CI: 1.029–1.060, *p* < 0.001), PLR (HR: 1.002, 95% CI: 1.001–1.003, *p* = 0.002), and the HALP score (HR: 0.827, 95% CI: 0.740–0.924, *p* = 0.001) were found to be statistically significant. In the multivariate Cox proportional hazards regression analysis, adjusted for gender, distant metastasis, dichotomized T stage, dichotomized N stage, and adjuvant chemotherapy status, only age (HR: 1.046, 95% CI: 1.030–1.062, *p* < 0.001) and the HALP score (HR: 0.984, 95% CI: 0.971–0.998, *p* = 0.025) remained independent predictors of mortality in patients with colon cancer. Bootstrap internal validation with 1000 resamples (bias-corrected and accelerated [BCa] 95% CI: −0.031 to −0.007) supported the stability of this result (bootstrap *p* = 0.025).

Table 2 presents the univariate and multivariate Cox regression analyses of predictors for mortality in patients with colon cancer.

**Figure 2:**
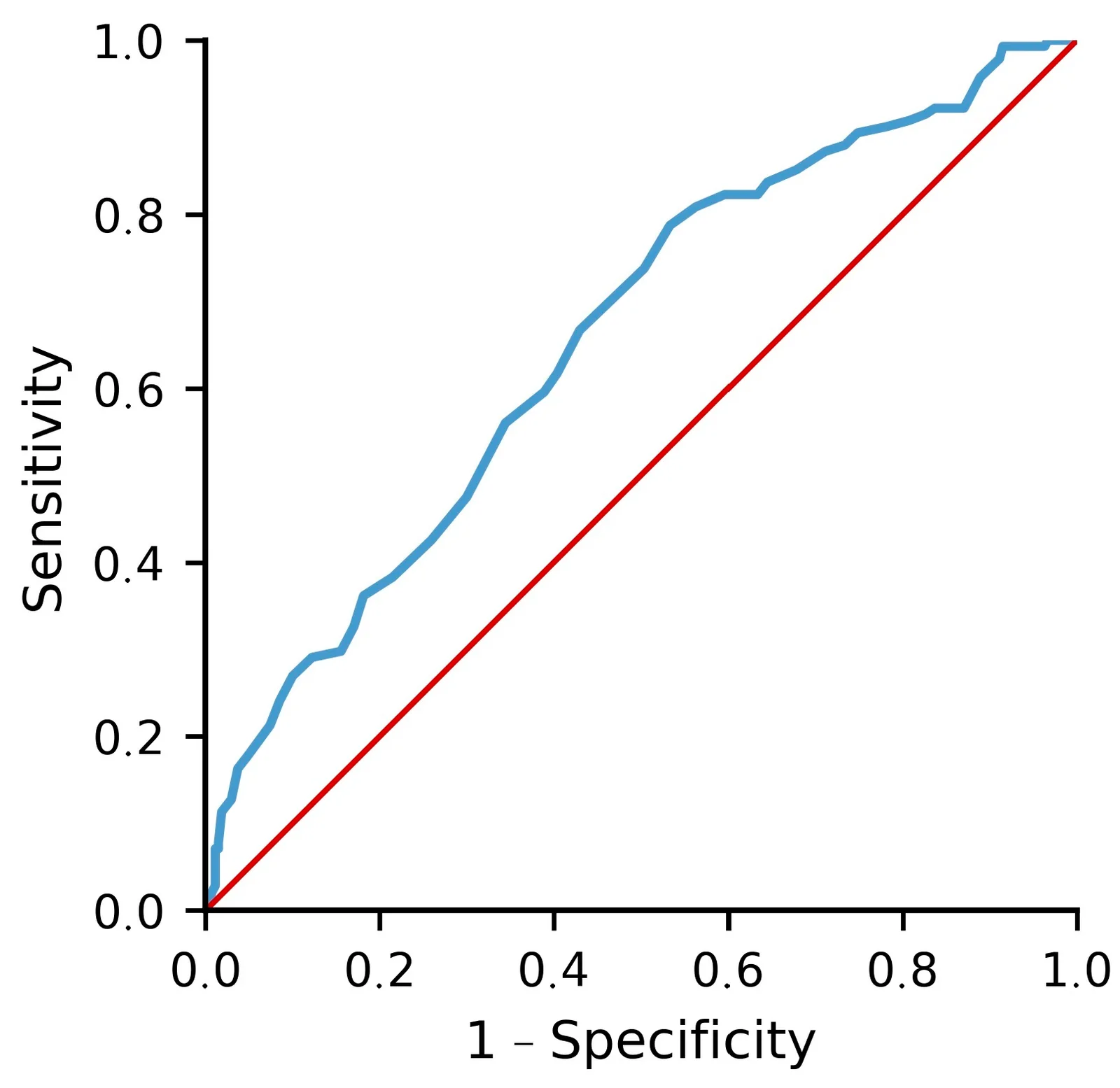
Receiver operating characteristic (ROC) curve for age as a predictor of mortality in patients with colon cancer. Optimal cut-off: 61.5 years (AUC = 0.657, 95% CI: 0.602–0.712, *p* < 0.001); sensitivity 79%, specificity 53%. The blue line represents the ROC curve for age; the red diagonal line represents the reference line of no discrimination (AUC = 0.5).

**Figure 3:**
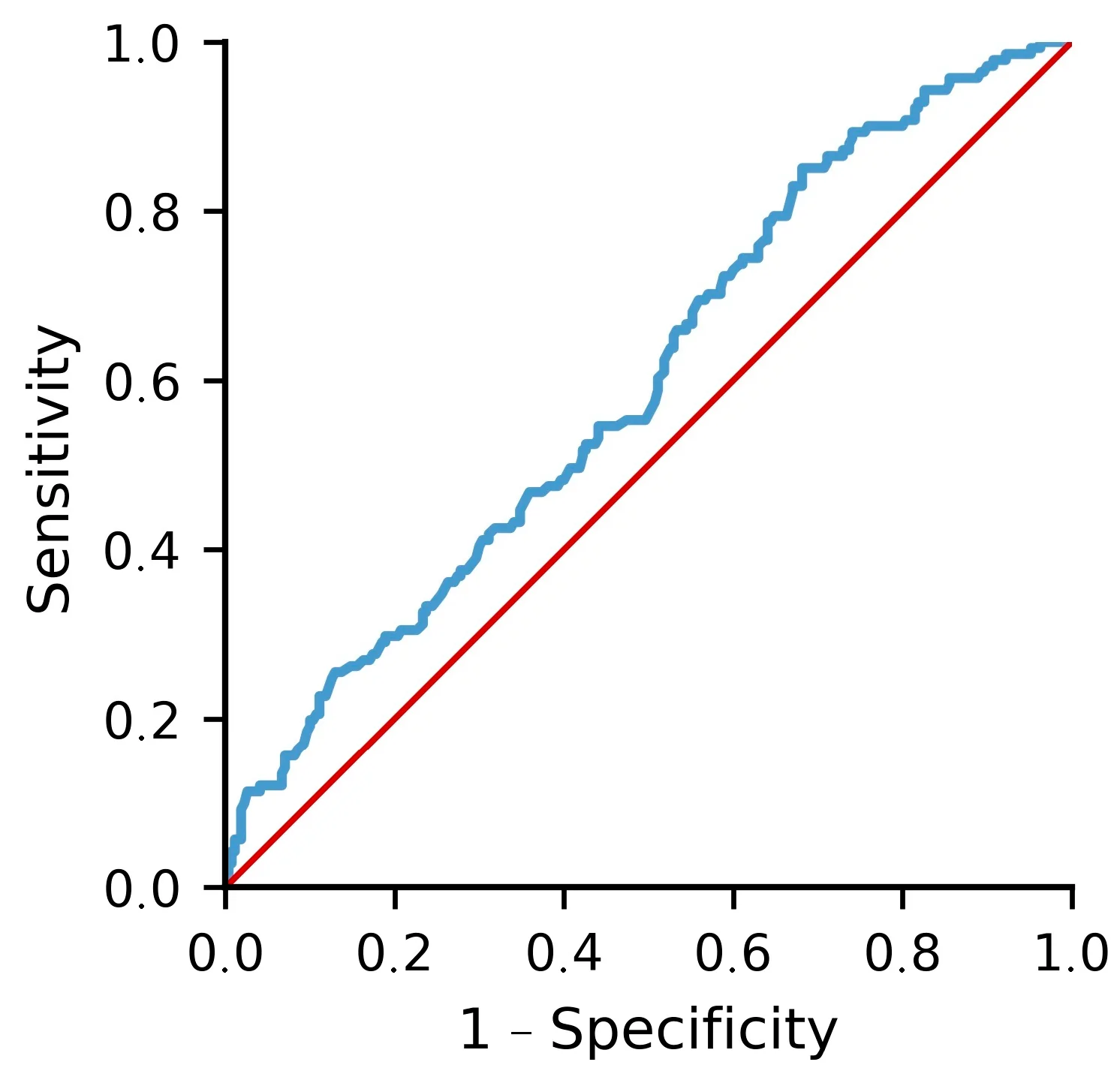
Receiver operating characteristic (ROC) curve for the standardized Hemoglobin, Albumin, Lymphocyte, and Platelet (HALP) score as a predictor of mortality in patients with colon cancer. Optimal cut-off (Youden index): 38.8 (AUC = 0.597, 95% CI: 0.540–0.654, *p* = 0.001); sensitivity 85%, specificity 32%. The blue line represents the ROC curve for the HALP score; the red diagonal line represents the reference line of no discrimination (AUC = 0.5).

The ability of independent predictors to predict mortality in patients with colon cancer is shown in Table 3. Age was able to predict mortality in patients with colon cancer with a cut-off value of 61.5 years (AUC: 0.657, 95% CI: 0.602–0.712, *p* < 0.001), with 79% sensitivity and 53% specificity (Figure 2), while the universally standardized HALP score, optimized via the Youden Index, predicted mortality with a cut-off value of 38.8 (AUC: 0.597, 95% CI: 0.540–0.654, *p* = 0.001), with 85% sensitivity and 32% specificity (Figure 3).

**Figure 4:**
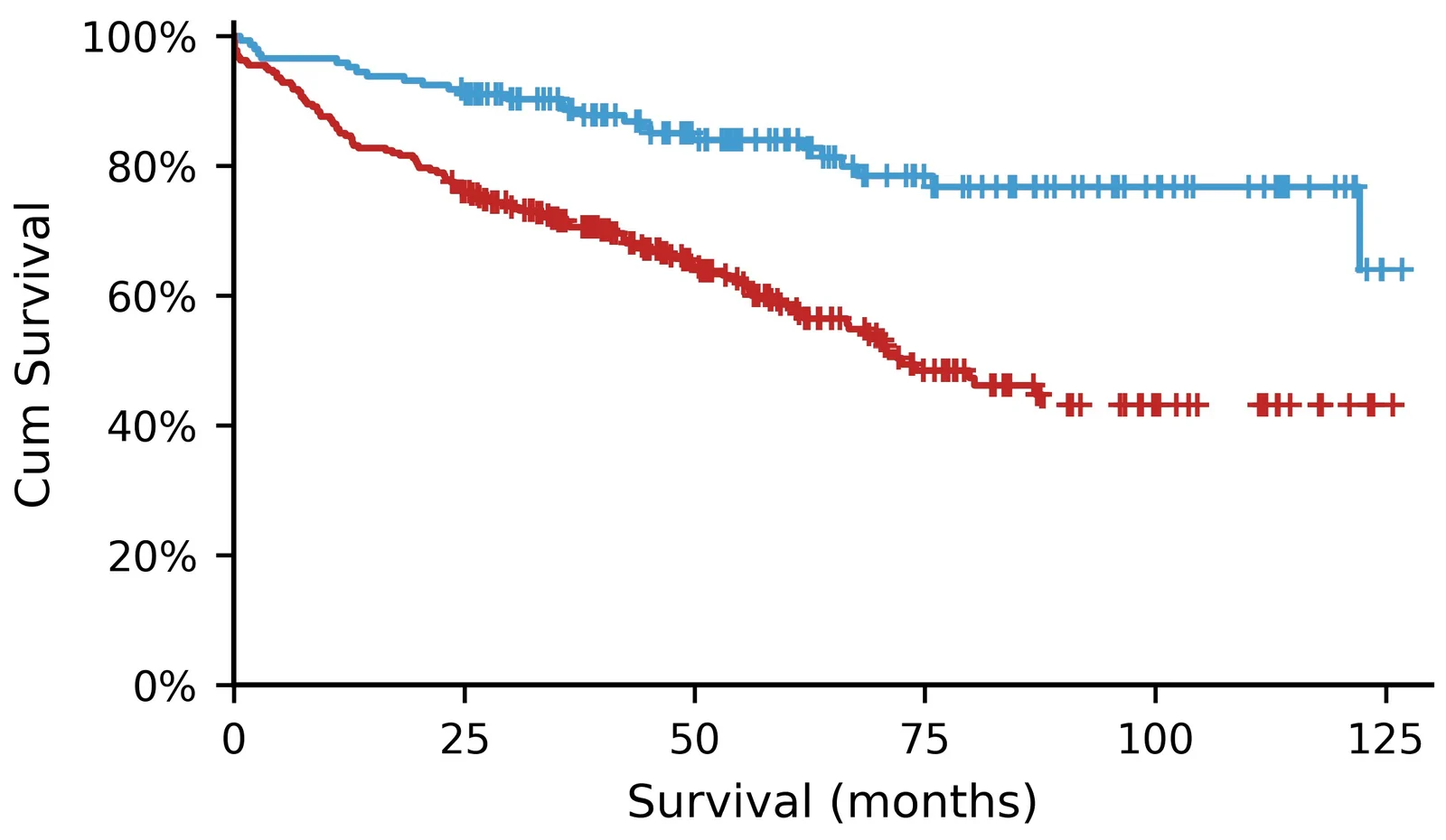
Kaplan–Meier survival curves for mortality in patients with colon cancer stratified by age (<61.5 vs. ≥61.5 years). Mortality was 18.6% in the younger group (mean survival: 108.8 months, 95% CI: 101.0–116.6) versus 42.9% in the older group (mean survival: 75.9 months, 95% CI: 69.3–82.6); log-rank χ^2^ = 25.878, *p* < 0.001. The blue line represents patients aged < 61.5 years; the red line represents patients aged ≥ 61.5 years.

Table 4 and Table 5 show the Kaplan–Meier survival analyses of the age and HALP score for mortality in patients with colon cancer, respectively. One hundred and forty-five (35.3%) patients were aged < 61.5 years, while 266 of them (64.7%) were aged ≥ 61.5 years. In the group aged < 61.5 years, the mortality rate was 18.6% (survival time: 108.8 (95% CI: 101.0–116.6) months), and in the group aged ≥ 61.5 years, it was 42.9% (survival time: 75.9 (95% CI: 69.3–82.6) months); this difference was statistically significant (log-rank chi-square: 25.878, *p* < 0.001) (Figure 4).

**Figure 5:**
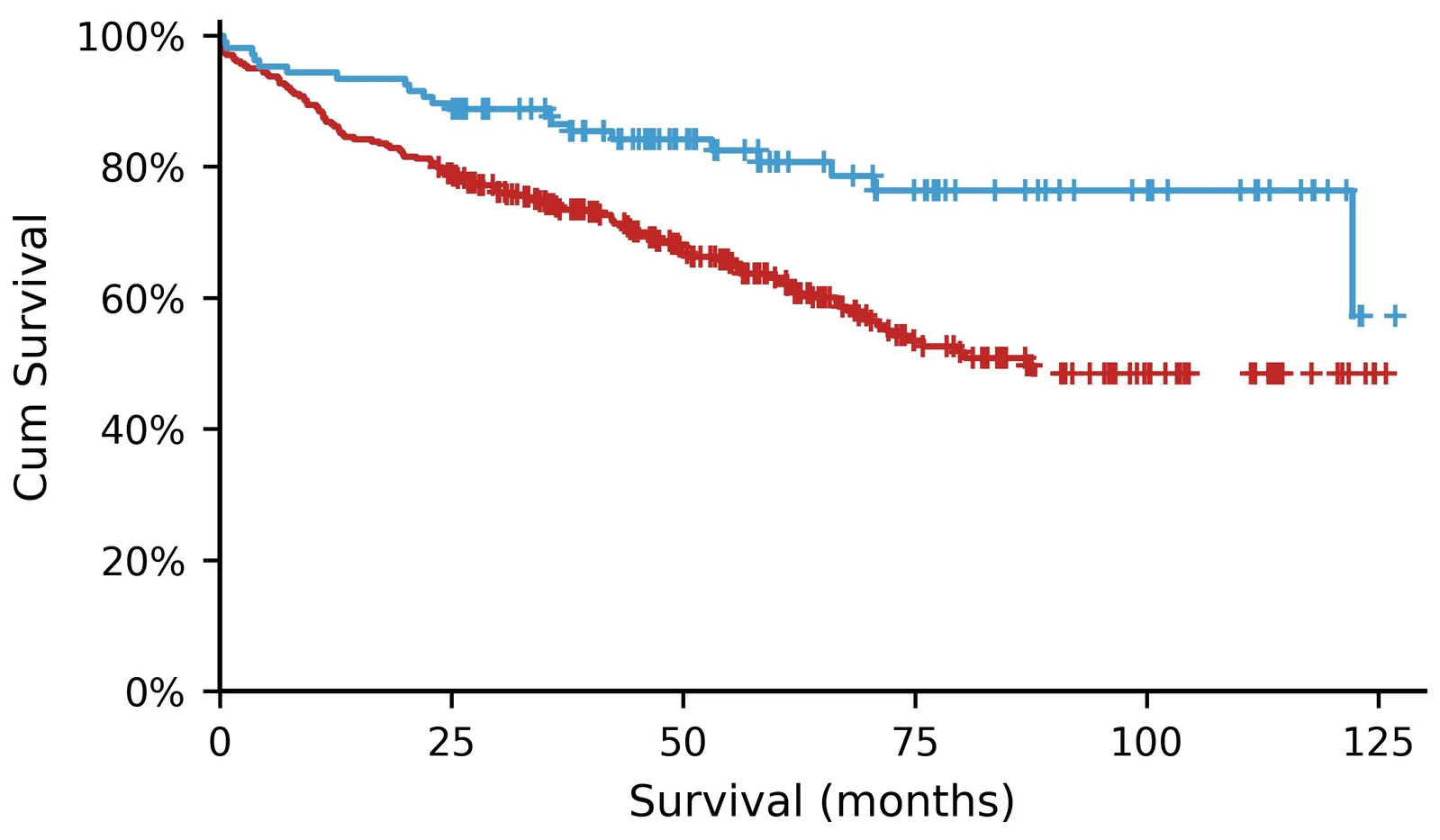
Kaplan–Meier survival curves for mortality in patients with colon cancer stratified by the standardized HALP score (>38.8 vs. ≤38.8). Mortality was 19.6% in the high-HALP group (mean survival: 103.94 months, 95% CI: 95.27–112.6) versus 39.5% in the low-HALP group (mean survival: 83.58 months, 95% CI: 77.06–90.1); log-rank χ^2^ = 12.423, *p* < 0.001. The blue line represents patients with a HALP score > 38.8; the red line represents patients with a HALP score ≤ 38.8.

Following the uniform database conversion, 107 (26%) patients had a standardized HALP score > 38.8, while 304 (74%) patients had a HALP score ≤ 38.8. In the group with a HALP score > 38.8, the mortality rate was 19.6% (mean survival time: 103.94 (95% CI: 95.27–112.6) months), and in the group with a HALP score ≤ 38.8 it was 39.5% (mean survival time: 83.58 (95% CI: 77.06–90.1) months); this survival divergence remained highly statistically significant (log-rank chi-square: 12.423, *p* < 0.001) (Figure 5).

## 4. Discussion

The present study investigated the prognostic significance of routinely accessible inflammatory, nutritional, and clinical parameters in patients undergoing surgery for colon cancer. The main findings of this study were that advanced age and a lower preoperative HALP score were independently associated with increased mortality. Furthermore, patients with HALP scores higher than 38.8 demonstrated significantly longer overall survival than those with lower scores, which suggests that the preoperatively measured universally standardized HALP score may be useful as a clinically simple prognostic biomarker in patients with colon cancer.

A higher age has been reported to be associated with reduced overall survival in colon cancer patients [11]. With increasing age, the physiological reserves decrease, comorbidities increase, and the immune function is negatively affected. All of these may contribute to poor long-term outcomes and lower tolerance to treatments. In this study, consistent with the literature, a higher age was confirmed as an independent negative predictor of overall survival. Patients aged ≥ 61.5 years experienced significantly worse survival than younger patients. This age threshold likely reflects immunosenescence, the age-related decline in immune function. With age, thymic output of naive T cells decreases, bone marrow reserve diminishes, and chronic low-grade inflammation (inflammaging) becomes more prominent. These changes may reduce the ability to mount an effective antitumor immune response and lower tolerance to physiological stress and adjuvant therapy, contributing to worse survival [17].

The most important finding of this study was the prognostic value of the HALP score on the overall survival in patients with colon cancer. The HALP score is a composite biomarker that includes four parameters, hemoglobin, albumin, lymphocyte count, and platelet count, reflecting the nutritional status, anemia, tissue oxygenation, systemic inflammation, antitumor immune activity, and immune competence, which represent cancer–host interaction [13]. Since prognosis is influenced not only by tumor characteristics but also by host status, this composition may provide a more comprehensive assessment compared with isolated parameters. However, although the standardized HALP score showed high sensitivity (85.1%) for predicting overall mortality, its specificity was low (31.9%) and the AUC was modest (0.597). This suggests that a HALP score above 38.8 is a reasonably reliable marker of favorable survival, but a low score is less specific and may also reflect non-oncological factors [13]. Therefore, the HALP score should not be used alone as a diagnostic or predictive tool; it should be combined with standard staging systems such as AJCC anatomical staging.

Clinically, a HALP score below 38.8 usually reflects several unfavorable host factors occurring together rather than in isolation: mild anemia, reduced albumin, relative lymphopenia, and reactive thrombocytosis. This combined pattern points to a host that is both nutritionally depleted and immunologically compromised, which is consistent with the mechanisms discussed below.

Several mechanisms may explain the association between lower HALP scores and poorer survival outcomes. Chronic inflammation within the tumor microenvironment promotes cancer progression by remodeling the local immune cell composition [18]. Hypoalbuminemia was found to be associated with malnutrition and chronic systemic inflammation and has been linked to adverse survival in colorectal cancer patients [19]. Tumor-derived pro-inflammatory cytokines such as IL-6 and TNF-α suppress hepatic albumin synthesis and increase vascular permeability, which together lower serum albumin [20]. Lymphopenia decreases the antitumoral response ability of the immune system, which reduces the density and cytolytic activity of tumor-infiltrating lymphocytes needed for effective antitumor immunosurveillance [21]. Anemia may reflect advanced disease and malnutrition [22,23] as tumor-associated anemia can cause intratumoral hypoxia, which stabilizes HIF-1α and promotes epithelial–mesenchymal transition and resistance to apoptosis and chemotherapy [24]. Platelets may facilitate tumor growth and dissemination by releasing angiogenic factors such as VEGF and forming a protective microthrombus shield around circulating tumor cells that limits natural killer cell-mediated lysis during hematogenous spread [25]. Consequently, lower HALP scores may identify patients with a combination of malnutrition, immune dysfunction, and systemic inflammation, all of which contribute to unfavorable survival outcomes.

Our findings are in agreement with recent studies investigating HALP scores in patients with colon cancer. A study including 640 patients with colon cancer showed that a lower HALP score, with a cut-off value of 15, was associated with shorter overall survival [26]. Another study by Xie et al. demonstrated that a lower HALP score, with a cut-off value of 32.4, was significantly linked with progression-free survival, overall survival, and sarcopenia in colorectal cancer [27].

In the multivariate Cox regression model, only the HALP score and age were independent predictors of overall survival, while the PLR, NLR, GRIM score, and De Ritis score did not reach statistically significant values. However, in the univariate analysis, the PLR reached a significant value. This does not mean the other markers have no prognostic value at all. They may simply reflect host immunity through a narrower, two-cell axis, whereas the HALP score combines lymphocyte and platelet counts with hemoglobin and albumin, capturing a wider range of host status. Hemoglobin and albumin are not only nutritional markers; they support tissue oxygenation and limit tumor-related catabolism, which helps preserve immune cell function. The GRIM score was likely not significant in our cohort because most patients had baseline values below its diagnostic thresholds (NLR ≤ 6 and LDH ≤ 250 U/L), giving a median score of zero across all subgroups. Overall, the HALP score may reflect host immune and physiological status more broadly than markers based on a single cell ratio, which may explain its predictive power in our multivariate analysis. This finding was confirmed after adjusting for gender, distant metastasis, dichotomized T and N stage, and adjuvant chemotherapy status, and after internal validation with 1000 bootstrap resamples. That the HALP score remained significant after this adjustment suggests it captures host-related prognostic information not reflected in anatomical staging alone [13].

An interesting finding of the present study was that the pathological T stage and N stage were not significantly associated with mortality in either the univariate or multivariate analyses. This is inconsistent with the established prognostic role of TNM staging and likely reflects the structure of our cohort rather than a true absence of prognostic value. The pathological stage distribution was highly skewed rather than evenly spread: more than 70% of patients had T3 disease, and over 83% were classified as N0. Even after combining T3 and T4 into a single “advanced T” category for the multivariable model, this distribution remained too imbalanced to reach statistical significance, which likely reduced the statistical power needed to detect stage-related survival differences in the Cox model. The proportion of metastatic patients was also very low (2.4%), further limiting the impact of advanced-stage disease on the overall survival analyses. In addition, patients presenting with emergency conditions such as obstruction or perforation were excluded; this exclusion likely removed tumors with more aggressive, inflammatory biology and poorer prognosis [28]. While necessary to keep baseline hematological values stable, it produced an elective cohort with generally milder tumor biology, which may have reduced the prognostic power of TNM staging in this analysis.

This exclusion also has implications for how the HALP score should be applied in practice. In acute emergency settings such as perforation or obstruction, the sudden systemic inflammatory response can rapidly lower lymphocyte and albumin levels and raise platelet counts, which would make the HALP score unstable and less representative of a patient’s true baseline status. The score is therefore likely most reliable in stable, elective patients, and its performance in emergency presentations remains to be studied separately.

Several limitations should be acknowledged. First, the retrospective single-center design may have introduced selection bias and may limit the generalizability of the findings. Second, hereditary cancer syndrome status (such as Lynch syndrome or familial adenomatous polyposis), and microsatellite instability (MSI) or KRAS/NRAS/BRAF mutation testing were unavailable for most patients in this historical cohort. Since MSI-high tumors are typically associated with dense lymphocytic infiltration, this missing molecular information could have influenced circulating lymphocyte counts and, in turn, the HALP score, in addition to carrying a distinct prognosis. Third, the modest specificity (31.9%) and area under the curve (0.597) limit its use as a primary diagnostic index; it should be interpreted as a secondary screening tool. In addition, the optimal HALP score cut-off value may differ between patient populations and, therefore, requires external validation. Despite these limitations, the present study includes a relatively large cohort of surgically treated colon cancer patients and provides additional evidence supporting the prognostic significance of the HALP score for overall survival in colon cancer.

## 5. Conclusions

In conclusion, the preoperative universally standardized HALP score is an independent predictor of overall survival in patients undergoing elective surgery for colon cancer. While its low statistical specificity precludes its use as a primary standalone prognostic factor, its high sensitivity makes it a simple, low-cost, widespread, and readily available immune–nutritional biomarker, meaning the HALP score may contribute to supplementary preoperative risk stratification and prognostic assessment in routine clinical practice. In this way, patients with low HALP scores may benefit from nutritional optimization, closer postoperative surveillance, and individualized management strategies. Future prospective multicenter studies are warranted to validate these findings and to determine the optimal cut-off value and the optimal integration of HALP into clinical decision-making algorithms in colon cancer.

## Figures and Tables

**Table 1:** Demographics, clinical, and laboratory data of whole study group and subgroups according to survival status ^x^.

	Study Group (*n* = 411)	Survival Group (*n* = 270, 65.7%)	Mortality Group (*n* = 141, 34.3%)	*p*
Age, years	65 (56–72)	63 (53–71)	69 (62–77)	**<0.001**
Gender, male	287 (69.8)	191 (70.7)	96 (68.1)	0.578
Localization				0.394
Right	283 (68.9)	185 (68.5)	98 (69.5)	
Transverse	7 (1.7)	3 (1.1)	4 (2.8)	
Left	121 (29.4)	82 (30.4)	39 (27.7)	
T stage				0.271
Tis	3 (0.7)	2 (0.7)	1 (0.7)	
1	6 (1.5)	5 (1.9)	1 (0.7)	
2	54 (13.1)	38 (14.1)	16 (11.3)	
3	297 (72.3)	198 (73.3)	99 (70.2)	
4	51 (12.4)	27 (10)	24 (17)	
N stage				0.653
0	342 (83.2)	229 (84.8)	113 (80.1)	
1	47 (11.4)	28 (10.4)	19 (13.5)	
2a	18 (4.4)	11 (4.1)	7 (5)	
2b	4 (1)	2 (0.7)	2 (1.4)	
M stage				0.083
0	401 (97.6)	266 (98.5)	135 (95.7)	
1	10 (2.4)	4 (1.5)	6 (4.3)	
TNM stage				0.399
0	3 (0.7)	2 (0.7)	1 (0.7)	
1	57 (13.9)	40 (14.8)	17 (12.1)	
2	276 (67.2)	184 (68.1)	92 (65.2)	
3	65 (15.8)	40 (14.8)	25 (17.7)	
4	10 (2.4)	4 (1.5)	6 (4.3)	
Postoperative chemotherapy	108 (26.3)	67 (24.8)	41 (29.1)	0.351
Indirect bilirubin (mg/dL)	0.17 (0.14–0.22)	0.18 (0.14–0.22)	0.17 (0.15–0.22)	0.506
Direct bilirubin (mg/dL)	0.15 (0.13–0.29)	0.15 (0.13–0.28)	0.16 (0.13–0.3)	0.266
Total bilirubin (mg/dL)	0.34 (0.24–0.65)	0.33 (0.24–0.62)	0.34 (0.24–0.8)	0.351
LDH (U/L)	200 (177–224)	196 (172–224)	201 (186–225)	0.131
Albumin (g/L)	40 (38–43)	40 (37–43)	40 (38–42)	0.102
Total protein (g/L)	62 (53–68)	62 (52–68)	63 (54–71)	0.715
ALT (U/L)	22 (14–37)	22 (14–37.5)	23 (16–38)	0.792
AST (U/L)	23 (16–38)	21 (15–37)	24 (17–40)	0.227
De Ritis ratio	1 (0.63–1.61)	1.05 (0.63–1.7)	0.95 (0.6–1.54)	0.373
CEA (ng/mL)	8.81 (4.42–25.18)	7.6 (4.45–18.2)	12.6 (4.04–38.1)	0.177
CA 19-9 (U/mL)	12.9 (6.63–28.5)	13 (6.76–27.25)	12.7 (6.45–34.85)	0.766
Neutrophil count (10^9^/L)	4.1 (3.2–5.3)	4.1 (2.49–5.3)	4.1 (3.2–5.3)	0.813
Lymphocyte count (10^9^/L)	1.78 (1.36–2.41)	1.84 (1.38–2.42)	1.77 (1.25–2.34)	0.141
Platelet count (10^9^/L)	295 (237–360)	292 (229–362.25)	299 (245–360)	0.071
Hemoglobin (g/L)	114 (104–128)	117 (104–130)	112 (103–123)	**0.007**
NLR	2.29 (1.6–3.37)	2.27 (1.51–3.34)	2.35 (1.7–3.41)	0.282
PLR	156.61 (111.18–226.45)	156.22 (105.24–222.78)	158.73 (129.45–242.52)	**0.033**
GRIM score	0 (0–0)	0 (0–0)	0 (0–0)	0.711
HALP score	28.8 (20.5–40.3)	29.6 (21.3–45.5)	27 (17.7–35)	**0.001**
Length of hospital stay, days	13 (10–17)	12 (10–17)	14 (10–17)	0.144
Survival, months	48.57 (28–73.5)	58.08 (39.22–84.31)	23.23 (9.03–47.89)	**<0.001**

^x^ Results were given as median (25–75 interquartile range) or frequency (percentages). Statistically significant *p* values were shown in bold. LDH: Lactate dehydrogenase, ALT: Alanine aminotransferase, AST: Aspartate aminotransferase, CEA: Carcinoembryonic antigen, CA19-9: Cancer antigen 19-9, NLR: Neutrophil to lymphocyte ratio, PLR: Platelet to lymphocyte ratio, GRIM: Gustave Roussy Immune, HALP: Hemoglobin, albumin, lymphocyte, and platelet.

**Table 2:** Univariate and multivariate Cox regression analyses of predictors for mortality in patients with colon cancer.

	Univariate Analysis				Multivariate Analysis			
		95% CI				95% CI		
	HR	Lower	Upper	*p*	HR	Lower	Upper	*p*
Age	1.044	1.029	1.060	**<0.001**	1.046	1.030	1.062	**<0.001**
Gender, male	0.910	0.638	1.296	0.600	0.895	0.626	1.280	0.544
Localization					-	-	-	-
Right	1	-	-	-				
Transverse	2.051	0.754	5.576	0.159				
Left	0.902	0.622	1.307	0.584				
T stage								
Early T (Tis-T2)	1	-	-	-	1	-	-	-
Advanced T (T3–T4)	1.259	0.767	2.065	0.363	1.436	0.863	2.389	0.164
N stage								
0	1	-	-	-	1	-	-	-
1–2	1.373	0.907	2.078	0.133	0.579	0.287	1.168	0.127
M stage								
0	1	-	-	-	1	-	-	-
1	2.039	0.898	4.631	0.089	0.420	0.165	1.066	0.068
TNM stage								
0–2	1	-	-	-	-	-	-	-
3–4	1.401	0.941	2.088	0.097	-	-	-	-
Postoperative chemotherapy								
	1.211	0.842	1.742	0.303	1.428	0.741	2.752	0.287
De Ritis ratio	0.902	0.741	1.098	0.304	-	-	-	-
CEA	1.001	0.999	1.004	0.257	-	-	-	**-**
CA 19-9	1	0.997	1.002	0.880	-	-	-	-
NLR	1.085	0.981	1.200	0.111	-	-	-	-
PLR	1.002	1.001	1.003	**0.002**	1.001	0.999	1.002	0.378
GRIM score	1.073	0.744	1.549	0.706	-	-	-	-
HALP score	0.827	0.740	0.924	**0.001**	0.984	0.971	0.998	**0.025**

HR: Hazard ratio, CI: Confidence interval, CEA: Carcinoembryonic antigen, CA19-9: Cancer antigen 19-9, NLR: Neutrophil to lymphocyte ratio, PLR: Platelet to lymphocyte ratio, GRIM: Gustave Roussy Immune, HALP: Hemoglobin, albumin, lymphocyte, and platelet. Significant *p* values are in bold.

**Table 3:** The ability of independent predictors to predict mortality in patients with colon cancer.

	AUC	95% CI		*p*	Cut-Off Value	Sensitivity	Specificity
		Lower	Upper				
Age	0.657	0.602	0.712	**<0.001**	61.5	0.787	0.533
HALP score	0.597	0.540	0.654	**0.001**	38.8	0.851	0.319

Significant *p* values are in bold. AUC: Area under curve, CI: Confidence interval, HALP: Hemoglobin, albumin, lymphocyte, and platelet.

**Table 4:** Kaplan–Meier survival analysis of age for mortality in patients with colon cancer.

	Total (*n* = 411)	Age < 61.5 Years (*n* = 145, 35.3%)	Age ≥ 61.5 Years (*n* = 266, 64.7%)	Log-Rank Chi-Square	*p*
Mortality	141 (34.3)	27 (18.6)	114 (42.9)	25.878	**<0.001**
Survival (months)	89.3 (95% CI: 83.7–94.9)	108.8 (95% CI: 101.0–116.6)	75.9 (95% CI: 69.3–82.6)		

CI: Confidence interval. Statistically significant *p* values are shown in bold.

**Table 5:** Kaplan–Meier survival analysis of HALP score for mortality in patients with colon cancer.

	Total (*n* = 411)	HALP > 38.8 (*n* = 107, 26%)	HALP ≤ 38.8 (*n* = 304, 74%)	Log-Rank Chi-Square	*p*
Mortality	141 (34.3)	21 (19.6)	120 (39.5)	12.423	**<0.001**
Survival (months)	89.3 (95% CI: 83.7–94.9)	103.94 (95% CI: 95.27–112.6)	83.58 (95% CI: 77.06–90.1)		

HALP: Hemoglobin, albumin, lymphocyte, and platelet, CI: Confidence interval. Statistically significant *p* values are shown in bold.

## Data Availability

The datasets generated and/or analyzed during the current study are not publicly available due to patient privacy and ethical restrictions but are available from the corresponding author on reasonable request and with the permission of the institutional review board.

## Abbreviations

The following abbreviations are used in this manuscript:

AJCC	American Joint Committee on Cancer
ALT	Alanine aminotransferase
AST	Aspartate aminotransferase
AUC	Area under the curve
CA 19-9	Cancer antigen 19-9
CEA	Carcinoembryonic antigen
CI	Confidence interval
GRIM	Gustave Roussy Immune
HALP	Hemoglobin, albumin, lymphocyte, and platelet
HR	Hazard ratio
LDH	Lactate dehydrogenase
NLR	Neutrophil-to-lymphocyte ratio
PLR	Platelet-to-lymphocyte ratio
ROC	Receiver operating characteristic
SPSS	Statistical Package for the Social Sciences
TNM	Tumor, Node, Metastasis (Staging System)
